# Modifying Wicking Speeds in Paper-Based Microfluidic Devices by Laser-Etching

**DOI:** 10.3390/mi11080773

**Published:** 2020-08-14

**Authors:** Brent Kalish, Mick Kyle Tan, Hideaki Tsutsui

**Affiliations:** 1 Department of Mechanical Engineering, University of California, Riverside, CA 92521, USA; bkali001@ucr.edu (B.K.); mtan010@ucr.edu (M.K.T.); 2 Department of Bioengineering, University of California, Riverside, CA 92521, USA; 3 Stem Cell Center, University of California, Riverside, CA 92521, USA

**Keywords:** paper-based microfluidics, laser-etching, wicking speeds, faster wicking

## Abstract

Paper-based microfluidic devices are an attractive platform for developing low-cost, point-of-care diagnostic tools. As paper-based devices’ detection chemistries become more complex, more complicated devices are required, often entailing the sequential delivery of different liquids or reagents to reaction zones. Most research into flow control has been focused on introducing delays. However, delaying the flow can be problematic due to increased evaporation leading to sample loss. We report the use of a CO_2_ laser to uniformly etch the surface of the paper to modify wicking speeds in paper-based microfluidic devices. This technique can produce both wicking speed increases of up to 1.1× faster and decreases of up to 0.9× slower. Wicking speeds can be further enhanced by etching both sides of the paper, resulting in wicking 1.3× faster than unetched channels. Channels with lengthwise laser-etched grooves were also compared to uniformly etched channels, with the most heavily grooved channels wicking 1.9× faster than the fastest double-sided etched channels. Furthermore, sealing both sides of the channel in packing tape results in the most heavily etched channels, single-sided, double-sided, and grooved, wicking over 13× faster than unetched channels. By selectively etching individual channels, different combinations of sequential fluid delivery can be obtained without altering any channel geometry. Laser etching is a simple process that can be integrated into the patterning of the device and requires no additional materials or chemicals, enabling greater flow control for paper-based microfluidic devices.

## 1. Introduction

Paper-based microfluidic devices are an attractive platform for developing low-cost, point-of-care diagnostic tools [1,2,3]. Paper is a lightweight, disposable, and widely abundant material that wicks liquid through channels without the need for external pumps. The simplest paper-based devices are lateral flow devices, where liquid wicks in one direction along the paper strip. These are suitable for simple detection chemistries; however, for more complex reactions, devices that are more complicated are required, and this frequently entails the sequential delivery of different liquids or reagents to reaction zones.

In order to develop devices capable of handling complex, multi-step (automated) detection chemistries, a variety of different flow control techniques have been proposed and implemented [4,5,6,7]. In particular, the issue of sequential delivery is important. To achieve sequential delivery, one can manually deposit reagents at different times, or deposit them simultaneously and have them arrive at times dictated by the dimensions of their travel paths.

Fluid flow in paper is frequently described by the Lucas–Washburn equation [8,9], which models paper as a bundle of capillary tubes, and describes the position of the liquid front *L* being proportional to the square root of time *t*:

(1) $$L\left(t\right)=\sqrt{\frac{\gamma D_{c}\cos\left(\theta \right)t}{4µ}}$$

where *γ* is surface tension, *D_c_* is the effective pore diameter, *θ* is the contact angle between the liquid and the paper (often taken to be zero in a fully wetting material such as paper), and *µ* is the dynamic viscosity. While not physically accurate as to the actual microstructure of paper (i.e., the interconnected, tortuous spaces between fibers look nothing like a bundle of parallel capillaries) and while it ignores ambient conditions (e.g., temperature, relative humidity), the Lucas–Washburn model works reasonably well at predicting the flow [10]. Where the model fails is with channels with different or non-constant widths. In general, wider channels wick more quickly than narrower channels, but those with increasing widths result in a slowing of the wicking front [11,12] and conversely, those with decreasing widths have a faster wicking front velocity [13,14].

For sequential delivery, modifying channel lengths and widths is the easiest method [12,15,16], but is constrained by the device’s footprint and available sample volume, as longer and wider channels will require larger volumes. The use of external shunts for wicking can also provide delays by effectively increasing the wicking length of the channel [17].

Thus, in a channel of constant width, according to the Lucas–Washburn model, in order to either increase or decrease the wicking speed of a given liquid, there are only two parameters that can be changed: the effective pore diameter or the contact angle. The contact angle of wetted, wicking paper is generally taken to be a low, near zero value, so increasing the contact angle would result in a more hydrophobic, slower wicking paper. As a result, most research into flow control has been focused on introducing delays, through techniques such as the addition of soluble [18,19] or insoluble [20,21,22] barriers, or compression [23] (resulting in smaller pores). However, delaying the flow can be problematic due to increased evaporation leading to sample loss, which requires an increased sample volume to counter.

The other option—speeding flow—is challenging, as it is difficult to increase the effective pore diameter. Typically, the maximum wicking speed is tied to the material choice. To increase wicking speeds beyond the limits of a given substrate, most attempts have focused on having the fluid travel outside the paper in external capillaries, either by sandwiching the paper between polymer films [24,25], creating multi-ply structures [26,27,28], or by removing portions of the paper to create empty channels for the liquid to flow through [29,30,31,32,33]. These techniques can result in much faster wicking, as much of the liquid is able to bypass the paper matrix entirely. This is ideal for devices that require rapid transport of large volumes of liquid between different regions of the device, but such behavior may be problematic or unsuitable for devices that utilize immobilized detection reagents or use small liquid volumes.

The present study, on the other hand, demonstrates the use of a CO_2_ laser to uniformly etch the surface of the paper, increasing its effective pore diameter and thus its wicking speed. By adjusting the grayscale levels of the design etched onto the paper, the resulting wicking speeds can be increased. Further increasing the grayscale levels results in the upper layers of the paper being removed entirely, significantly reducing the overall cross-section of the paper. This results in slower wicking speeds. By selecting the appropriate etching level, the desired wicking behavior can be achieved.

## 2. Materials and Methods

### 2.1. Materials

Whatman #1 chromatography paper (GE Healthcare, Chicago, IL, USA) was used as the wicking paper in all experiments. Whatman #17 chromatography paper (GE Healthcare, Chicago, IL, USA) was used as sample pads for the demonstration device. The wicking fluids for the demonstration device were the following dyes: 5 mM Erioglaucine-disodium salt, 5 mM tartrazine, and 5 mM Allura red. Chemicals were obtained from Sigma-Aldrich (St. Louis, MO, USA). Bovine serum albumin, Texas Red was purchased from Thermo Fisher Scientific (Waltham, MA, USA).

### 2.2. Etching

Whatman #1 chromatography paper was cut out and etched using a 30 W CO_2_ laser cutter (Zing 16, Epilog Lasers, Golden, CO, USA). The laser raster settings were 13% power, 45% speed, 500 dpi, and Floyd–Steinberg dithering and the vector settings were 7% power, 60% speed, and 5000 Hz. These settings correspond to the highest values that reliably did not completely burn through the paper at 100% grayscale values. Etching levels were controlled by the grayscale value of the channel fill, ranging from 0% (pure white) to 100% (pure black).

### 2.3. Mass Loss

Mass loss was measured by weighing 50 × 50 mm squares etched at each grayscale value and comparing them to identically sized unetched squares. Squares of paper were cut to identical sizes using the CO_2_ laser. Ten squares for each condition were prepared and weighed using an analytical scale (Model TP-64, Denver Instrument, Bohemia, NY, USA).

### 2.4. Scanning Electron Microscopy (SEM)

The effect of the laser etching on the paper’s microstructure was examined via SEM using a Nova NanoSEM 450 (FEI, Hillsboro, OR, USA).

### 2.5. X-ray Photoelectron Spectroscopy (XPS)

The surface composition of etched and unetched papers were examined using a Kratos AXIS Ultra XPS (Manchester, UK). Paper samples were also washed by dipping in deionized water (DI H_2_O) and then air dried to eliminate soot formed by the cutting/etching process.

### 2.6. Wicking Experiments

#### 2.6.1. Vertical Wicking

The layouts of the channel arrays were designed using graphical software (CorelDraw, Corel Corporation, Ottawa, ON, Canada). Each channel was 3 mm wide and 40 mm long. Wicking experiments were performed in a humidity-controlled chamber (Model 5503-E, Electro-Tech Systems, Glenside, PA, USA) kept at 55% RH and 23 °C. Each channel array was mounted vertically to the testing rig and placed in the chamber.

**Figure 1:**
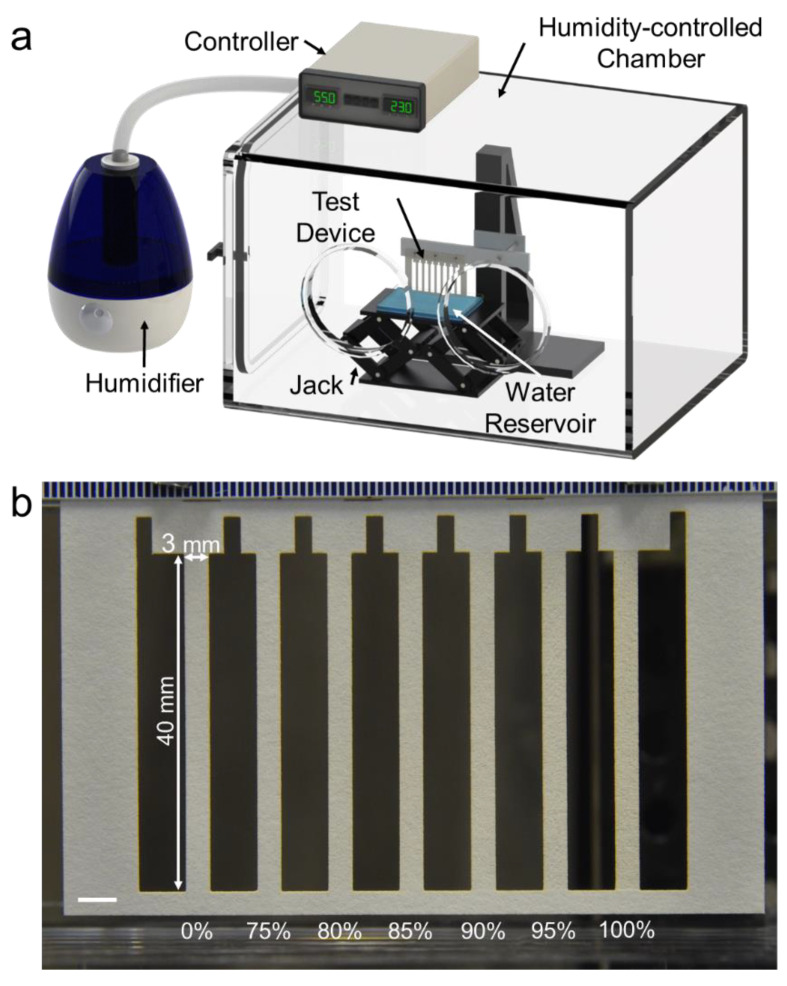
Wicking experimental setup. (**a**) Wicking was performed in a humidity-controlled chamber kept at 55% RH and 23 °C. DI H_2_O was wicked vertically in each channel to eliminate any potential effects caused by a backing. (**b**) Close-up view of the test device, with seven 3 mm wide, 40 mm long channels, etched at 0% (unetched), 75%, 80%, 85%, 90%, 95%, 100% grayscale values. Scale bar is 5 mm.

DI H_2_O in a reservoir was placed onto a laboratory jack (Model L-490, Thorlabs, Newton, NJ, USA) and then quickly raised to the bottom of the paper. The fluid fronts were recorded using a Nikon D5100 (Tokyo, Japan). Video files were processed into individual frames using Adobe Premier Pro CS6 (San Jose, CA, USA). The wicking distances were then recorded using ImageJ’s Manual Tracking plugin (ImageJ v1.52d). Figure 1 depicts the experimental wicking setup.

#### 2.6.2. Angled Wicking

**Figure A1:**
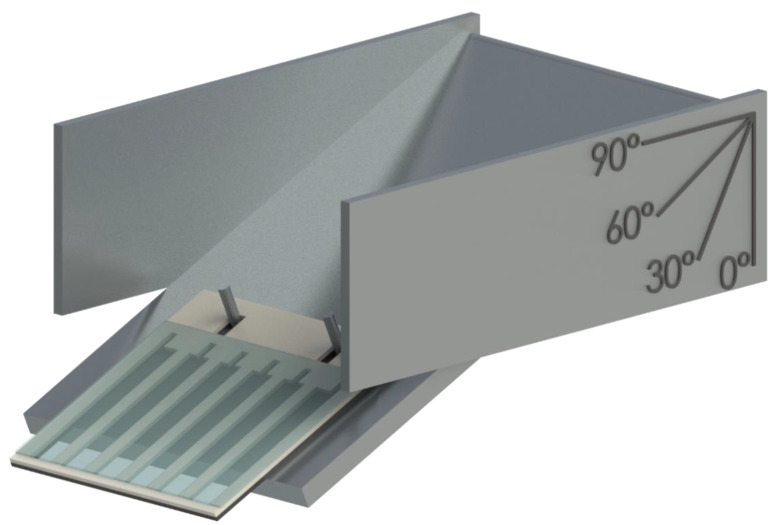
Angled wicking setup. The angled platform was held at the indicated angles by a removeable crossbar. The rig was mounted to the frame in the humidity chamber depicted in Figure 1. At 0°, 30°, and 60°, the water reservoir is raised on a jack to the bottom of the paper. For 90°, a saturated polyester sponge with an overhang was lowered by the jack until the overhang rested on the bottom edge of the paper.

Angled wicking experiments utilized the same chamber and jack with a modified testing rig that allowed the paper to be mounted at 0°, 30°, 60°, and 90° from the vertical. A depiction of this setup is in Figure A1. Wicking at 90° required the use of a saturated sponge to deliver DI H_2_O to the channels.

#### 2.6.3. Grooves

**Figure A2:**
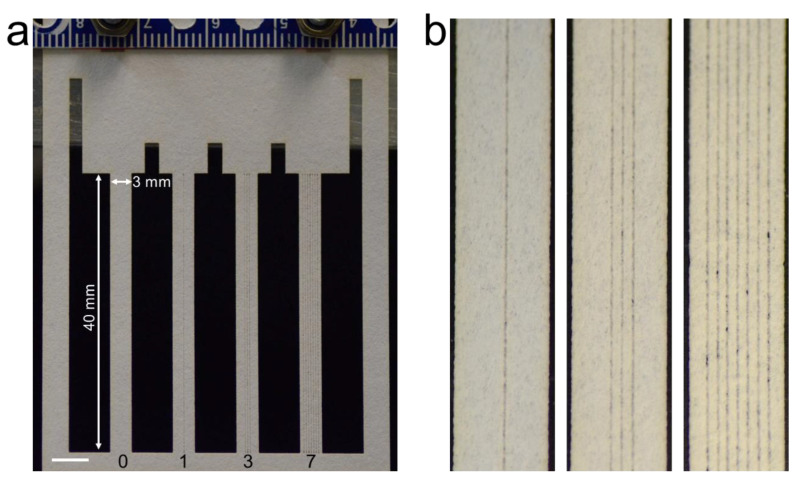
Close-up of the grooved channels. (**a**) The grooved test device, with four 3 mm wide, 40 mm long channels, etched with 0 grooves (unetched), 1, 3, and 7 grooves. Scale bar is 5 mm. (**b**) A close-up view of the grooves. The grooves are centered in the channels with 400 µm center-to-center spacing.

Grooves were etched into the paper using the same CO_2_ laser with the laser set to 5% power, 100% speed, and 5000 Hz. The grooves ran the length of the channels with a center-to-center spacing of 400 µm. A depiction of the grooved channels is in Figure A2.

#### 2.6.4. Taped Channels

The previously described angled wicking experiments were repeated with packing tape (Scotch Heavy Duty Shipping Packaging Tape, 3M, Two Harbors, MN, USA) sealing the front and back of each channel.

#### 2.6.5. Demonstration Device for Sequential Deliveries

**Figure A3:**
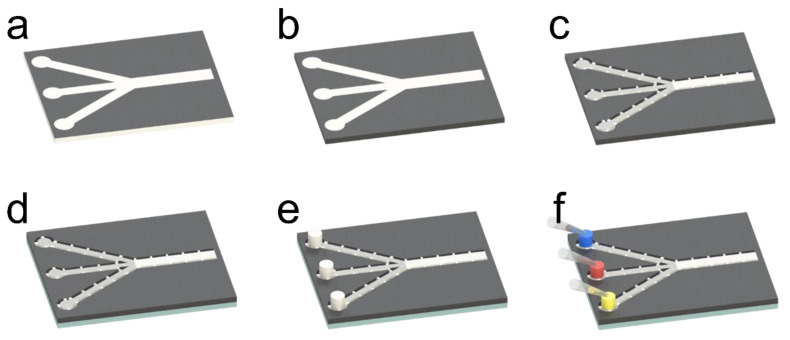
Fabrication process flow for demonstration device. (**a**) A wax layer defining the channels was printed using a solid wax printer onto Whatman #1 chromatography paper. (**b**) The wax was melted by placing the paper into an oven at 170 °C for 2 min. (**c**) The paper was etched and the channels were cut to final dimensions. The presence of the side-struts along the edge of the channels are for support, preventing the wet channels from buckling and lifting up from their backing. (**d**) The bottom side of the devices were coated with packing tape to form a backing. (**e**) Rounds of Whatman #17 chromatography paper were used as sample pads to regulate flow into the channels. (**f**) 100 µL of 5 mM dye was introduced into each channel simultaneously using a multi-channel pipette.

The demonstration device was first printed using a solid wax ink printer (ColorQube 8880DN, Xerox Corporation, Norwalk, CT, USA) and then placed in an oven (Model FD 53, Binder, Tuttlingen, Germany) at 170 °C for 2 min to melt the wax, forming hydrophobic barriers. After melting, the devices were cut and etched with the CO_2_ laser cutter. Devices were then backed with the packing tape and rounds of Whatman #17 chromatography paper were used as sample pads. The fabrication process flow of the devices is depicted in Figure A3. The wicking fluids were 100 µL of each of the following dyes: 5 mM Erioglaucine-disodium salt, 5 mM tartrazine, and 5 mM Allura Red. Channels were 3 mm wide with the two outer legs of equal length (~50 mm), and a shorter center channel (~40 mm).

### 2.7. Protein Immobilization

**Figure A4:**
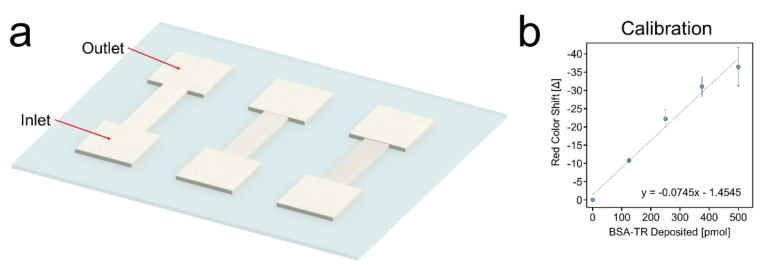
Protein transport test channels. (**a**) Each test strip had a 7 × 7 mm square inlet that lead to a 3 × 10 mm channel etched at either 0%, 50%, or 100%. In each inlet, 20 μL of either 50 or 100 μM BSA-TR was deposited and allowed to wick to the outlet. After 30 min, the red channel intensity of each outlet was compared to a blank to determine the red color shift and its corresponding BSA-TR concentration. (**b**) Calibration curve created from 7 × 7 mm squares deposited with a known quantity of BSA-TR. Data presented as mean ± SD. *n* = 3.

Solutions of bovine serum albumin conjugated with Texas Red (BSA-TR) were wicked along channels etched at various levels (0%, 50%, and 100%) to determine if the laser-etched paper inhibits protein transport. The test devices contained 7 mm square inlets and outlets connected by a 3 mm wide, 10 mm long channel (Figure A4). Images of the outlets were taken when dried, 30 min after initial BSA-TR deposition. Camera settings were: f/4.5, 1/320s, ISO-1600. The red color shifts (*RCS*(Δ)) of the outlets were measured by comparing the red channel intensity values of the BSA-TR outlets to those of blank outlets using the equation:

(2) $$RCS\;\left(\Delta \right)=R_{0}-R_{s}$$

where *R_s_* is the red intensity value of the deposited BSA-TR and *R_0_* is the red intensity value of a blank outlet. The red channel was used as it produced a greater shift than the blue channel as a result of the BSA-TR deposition. A calibration curve was prepared by depositing known molar quantities (0–500 pmol in 125 pmol increments) of BSA-TR onto 7 mm squares and measuring the red color shift associated with each quantity (Figure A4). From this calibration curve, the red color shift of the outlets could then be converted to a molar quantity and then determine the percentage of the deposited BSA-TR that reached the outlet [34].

## 3. Results and Discussion

### 3.1. Etching

**Figure 2:**
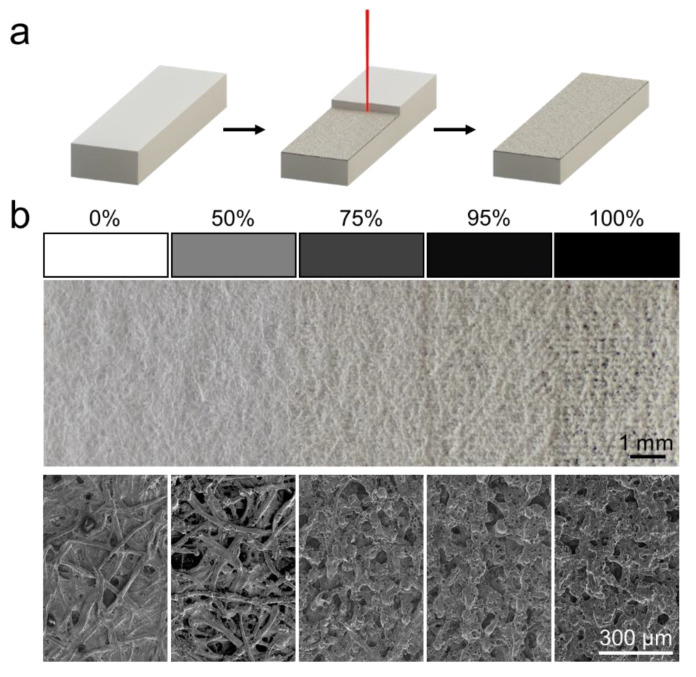
Laser-etched paper. (**a**) Schematic of the etching process. The etching process removes material from the top surface the paper. (**b**) Macro and SEM images of the etched paper surface. At the macro level, surface modification is not visible until 75%, but even at 50%, it is apparent that material has been removed from the surface in the SEM images.

The laser etching works by converting a grayscale image to a black and white image through dithering, where the average density of individual black dots will match the original grayscale value. The laser then rasters the image onto the paper, removing material from the surface of the paper. This is shown in Figure 2a. The effect of the etching process on the chromatography paper is shown in Figure 2b. While there are few visible differences at the macroscale between 0% (unetched) and 50% etched, the SEM image shows that at 50%, much of the material in between surface fibers has been removed. Surface remodeling becomes more significant as grayscale values increase, and at 100%, the paper is visibly perforated at the macroscale.

### 3.2. Mass Loss

**Figure 3:**
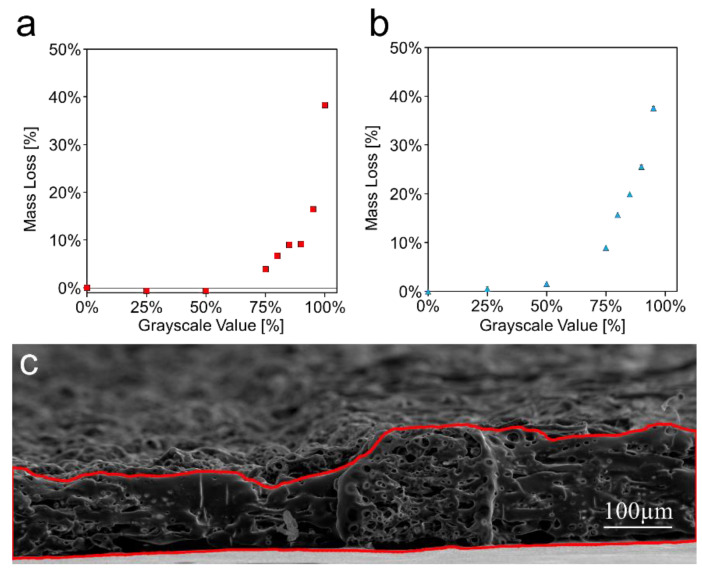
Mass loss caused by laser etching. (**a**) Percent mass loss at each etching level of single-sided etched paper. 50 × 50 mm squares of Whatman #1 chromatography paper were weighed after being etched at each grayscale value. *n* = 10. (**b**) Percent mass loss at each etching level of double-sided etched paper. Paper disintegrated when etched at 100% on both sides. Data presented as mean ± SD. *n* = 10. (**c**) SEM image showing the effect of etching on the cross-sectional area of the paper. The region on the left has been etched at 100% on a single side while the region on the right has not been etched.

While the SEM images show a noticeable difference in surface topology between the 0% and 50%, the actual mass loss at 50% is negligible, as seen in Figure 3a. The mass loss increases from approximately 4% at 75% grayscale to approximately 40% at 100% grayscale. Due to this, the grayscale values used for the wicking experiments were limited to values above 75%. Wicking was also investigated in paper etched on both sides. As shown in Figure 3b, the mass loss trend is quite similar as the single-sided etched paper, with very small mass losses below 75%. Paper etched on both sides at 100% disintegrated, so no value was able to be obtained. A cross-section view comparing the relative thicknesses of 100% etched vs. unetched paper can be seen in Figure 3c.

### 3.3. XPS

**Figure A5:**
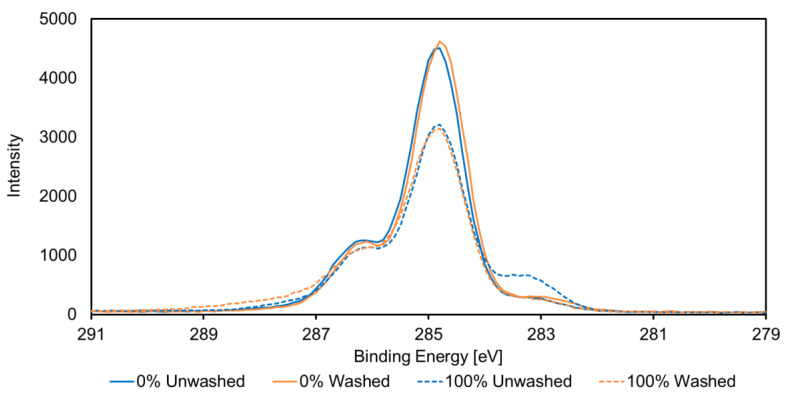
XPS Carbon 1s spectra of laser-etched Whatman #1 chromatography paper. Unetched paper (0%) was compared to paper etched at the highest grayscale value (100%). The washed paper was simply dipped into DI H_2_O and allowed to dry at room temperature.

XPS spectra analysis was performed to verify that the laser etching process had not altered the chemical composition of the surface. Laser-etched paper had an approximately 30% reduction in intensity of the carbon–carbon peak at 284.8 eV compared to the unetched paper and had a peak appear at approximately 283 eV that is suspected to correspond to soot, as it disappeared from samples that were rinsed in DI H_2_O after etching. The carbon C1 s spectrum can be found in Figure A5.

### 3.4. Wicking Experiments

#### 3.4.1. Vertical Wicking

**Figure 4:**
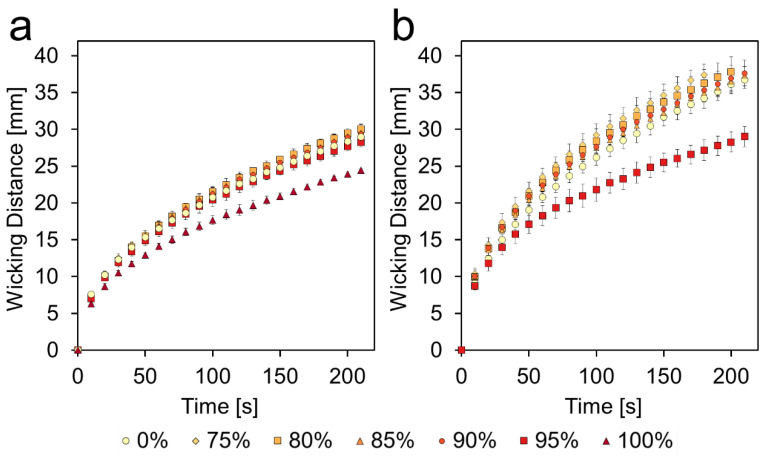
Wicking performance of single- and double-sided etched channels. (**a**) Average wicking distance vs. time at each etching level of single-sided etched paper. (**b**) Average wicking distance vs. time at each etching level of double-sided etched paper. Wicking was performed at 55% relative humidity and constant 23 °C. Data presented as mean ± SD. *n* = 3.

In general, the fastest wicking occurred in the 75−80% channels and wicking speeds decreased as the level of etching increased, ultimately resulting the 100% channel wicking even slower than the unetched channel. This can be seen in Figure 4a. This trend held true for the double-sided etched channels as well. As with the single-sided etched paper, the fastest double-sided channels were the 75–80% channel and speeds decreased as etching increased, up to 95%, which was much slower than the unetched channel. This can be seen in Figure 4b.

The above data suggest that there are two competing phenomena being observed. The first is an increase in wicking speeds due to a more porous surface and the second is a decrease in wicking speeds due to increased mass losses at higher etching levels, resulting in reduced cross-sectional areas.

**Figure 5:**
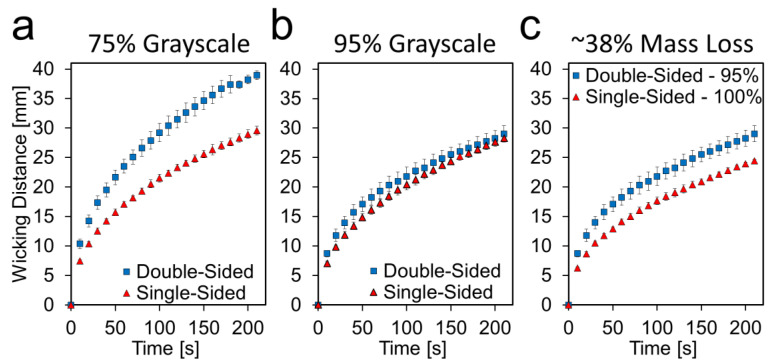
Average wicking distance vs. time at select etching levels of both single- and double-sided etched paper. (**a**) Single- and double-sided paper both etched at 75%. (**b**) Single- and double-sided paper both etched at 95%. (**c**) Single-sided paper etched at 100% and double-sided paper etched at 95% both result in ~38% mass loss, however the double-sided channels are faster, indicating that the speed increase is more than just a function of the total mass removed. Data presented as mean ± SD. *n* = 3.

Comparing the single- and double-sided channels directly, as in Figure 5, shows that at 75%, the double-sided channel is much faster than the single-sided channel, but as the etching level increases, that difference gets smaller and at 95%, the two channels behave quite similarly, despite the double-sided channel having twice the mass lost as the single-sided. This further suggests that the effects of the more highly porous surface are in opposition to the effects of the reduced cross-sectional area. This is evident in Figure 5c, which compares single-sided 100% and double-sided 95%. The two conditions have very similar mass loss percentages, ~38%, but the double-sided channel is noticeably faster than the single-sided channel.

#### 3.4.2. Demonstration Device

The data presented above are from vertical wicking, while the majority of paper-based microfluidic devices are used horizontally. In order to confirm that the vertical results were applicable to horizontal devices, a simple three-channel device was designed that demonstrates the technique’s utility in enabling sequential delivery.

**Figure 6:**
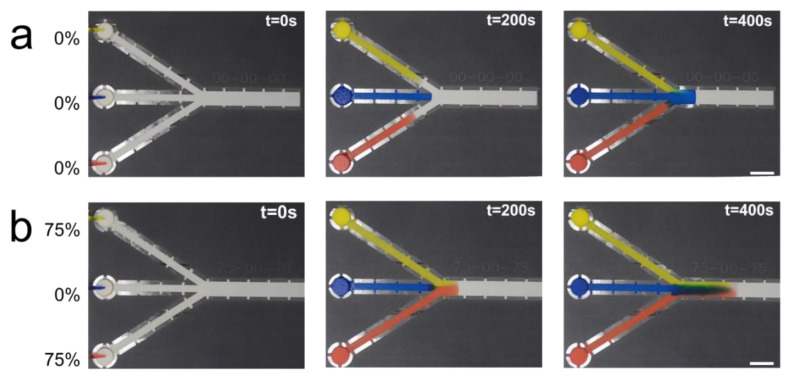
Time-lapse of the single-sided etched three-channel demonstration device. (**a**) All channels are unetched (0-0-0 device). The middle channel arrives at the intersection first, followed by the top and bottom channel that arrive nearly simultaneously. (**b**) The outer channels are etched at 75% and the middle channel is unetched (75-0-75 device). The outer channels arrive at the intersection simultaneously, while the middle channel takes longer. Scale bars are 10 mm.

Unmodified, the shorter central channel should reach the intersection first, followed by the simultaneous arrival of the longer outer channels. However, by etching different channels at different levels, not only can the relative arrival times of each channel be modified, but also their absolute wicking times. Figure 6 shows a time-lapse comparison of a device with unetched channels (0-0-0) and one with the two outer legs etched at 75% with the center leg remaining unetched (75-0-75). Time-lapse GIFs of both devices are available in the Supplementary Materials.

In the 0-0-0 device, all channels wicked the dyed solutions at the same speed; however, since the center channel was shorter, the blue solution reached the intersection first, followed by the simultaneous arrival of the red and yellow solutions. In the 75-0-75 device, the dye in the etched channels wicked much more quickly than in the unetched channel. The yellow and red dye reached the intersection simultaneously and arrived much sooner than the blue dye in the center unetched channel. Comparing the two different devices at t = 400 s, one can see the consequence of the different fluid arrival times on the degree of mixing of the different dyes. In the 0-0-0 device, after the intersection, the blue dye dominates and the other dyes slowly enter from the sides, whereas in the 75-0-75 device, the blue dye is washing the red and blue dye further along the channel.

#### 3.4.3. Angled Wicking

The wicking speeds in the demonstration device were, however, much faster than their corresponding vertical wicking speeds. The most likely cause for this discrepancy is the excess of liquid that races across the surface of the paper upon deposition. As noted before, traveling outside the paper is much faster than wicking through the paper. However, it is possible that vertical wicking is somehow hindered by gravity. To attempt to identify the role that the angle of the paper played on overall wicking speeds, the vertical wicking tests were repeated with additional samples at 30°, 60°, and 90° from the vertical (Figure A1).

**Figure 7:**
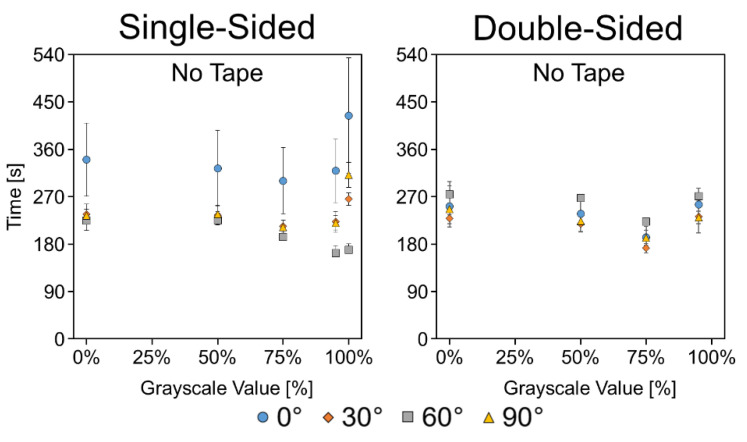
Angled wicking data for single- and double-sided etched channels without tape. Data presented as mean ± SD. *n* = 5.

**Figure A6:**
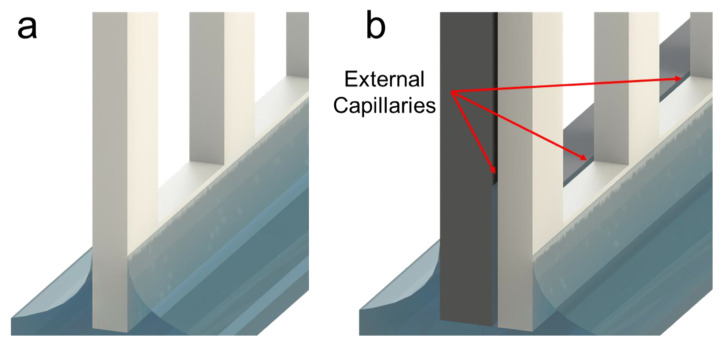
Depiction of why the angled wicking 0 degrees is faster than the previous data set. (**a**) Meniscuses form evenly on both sides of the paper. (**b**) External capillaries formed by the backing likely cause faster fluid delivery to the inlet of the channels.

**Figure A7:**
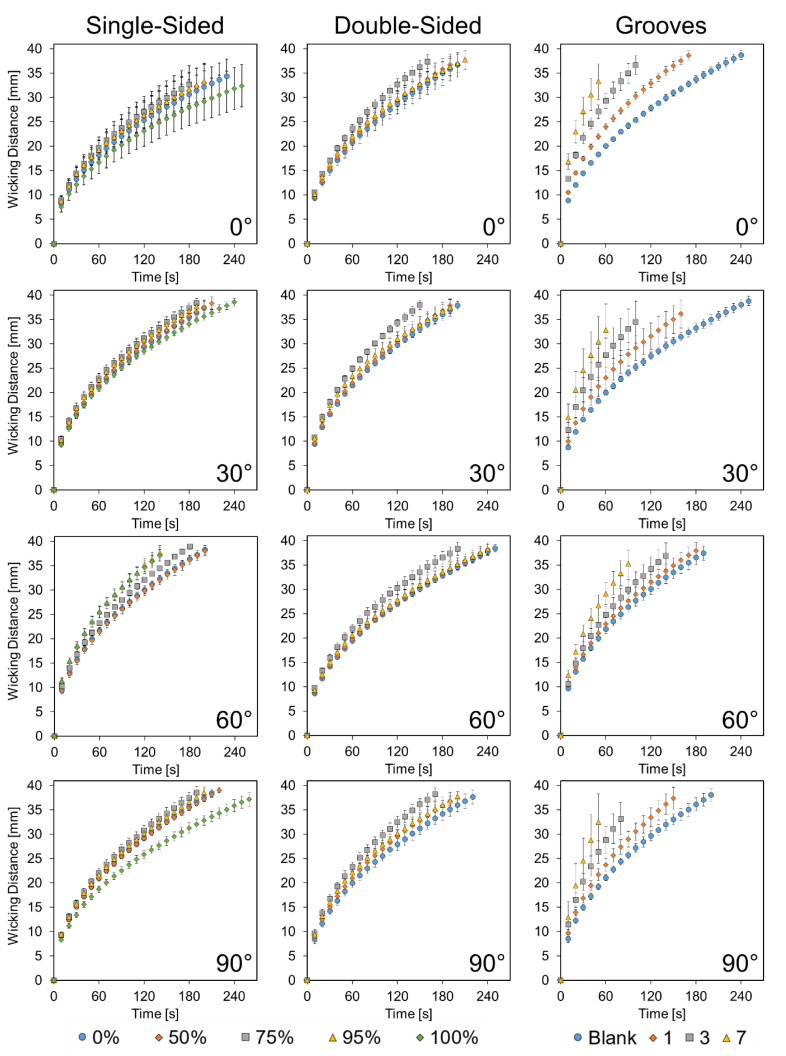
Distance vs. time plots for untaped angled wicking. Data presented as mean ± SD. *n* = 5.

Figure 7 shows the time it took liquid to wick the full 40 mm length of the channels at the various angles for both single- and double-sided etched paper. With respect to angle, there was no consistent trend among the different etching levels or single- vs. double-sided. However, the 0° channels wicked faster than the previous set of experiments. This is possibly due to the addition of a backing frame, required for support of the paper at increased angles (Figure A6). This frame results in the formation of external capillaries at the base of the channels, potentially allowing liquid faster access to the channels. A complete set of time vs. distance plots for the untaped channels are depicted in Figure A7, and *p*-values of the *t*-tests are listed in Table A1. Time-lapse GIFs of the untaped single- and double-sided etched channels at 0° are available in the Supplementary Materials.

#### 3.4.4. Taped Channels

**Figure 8:**
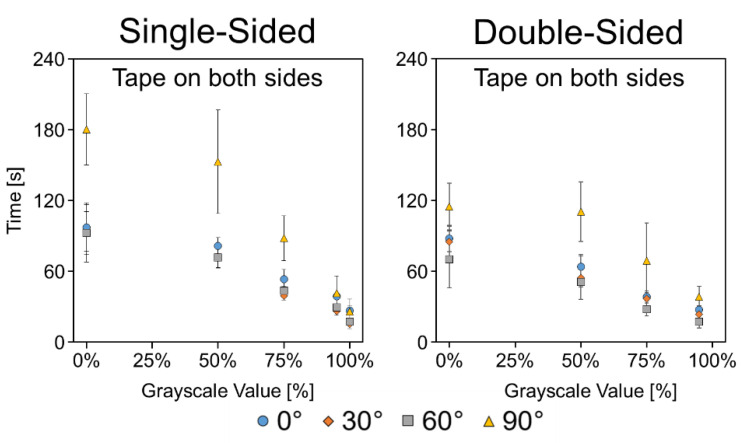
Angled wicking data for single- and double-sided etched channels with tape on both sides. Data presented as mean ± SD. *n* = 5.

**Figure A8:**
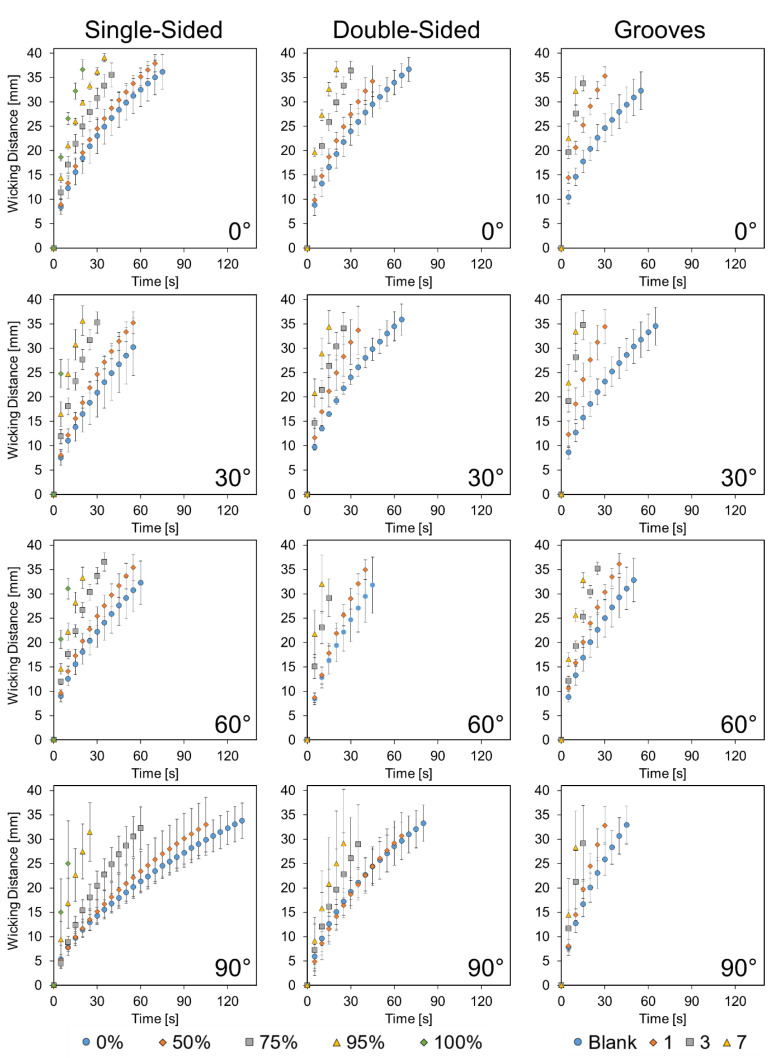
Distance vs. time plots for taped (double-sided) angled wicking. Data presented as mean ± SD. *n* = 5.

While the untaped channels showed inconsistencies at the various angles, once sealed with tape, the wicking times for the 0°, 30°, and 60° channels were remarkably consistent (Figure 8). Furthermore, they displayed a trend of increasing speed with increasing etching level, unlike the untaped channels which showed the fastest wicking with more moderate etching levels of 75–95%. The 90° channels were slower at lower etching levels, but this is likely due to the differences in how liquid was delivered to the horizontal channels. The horizontal channels could not be dipped in a liquid reservoir, so instead, a saturated polyester sponge was lowered onto the channels to deliver the liquid. The sponge method resulted in slow initial wicking as the sponge settled onto the paper, a slower process than the formation of the meniscus across the edge of the paper when dipping it into a reservoir. A complete set of time vs. distance plots for the taped channels are depicted in Figure A8, and *p*-values of the *t*-tests are listed in Table A2. Time-lapse GIFs of the taped single- and double-sided etched channels at 0° are available in the Supplementary Materials.

#### 3.4.5. Grooves

**Figure 9:**
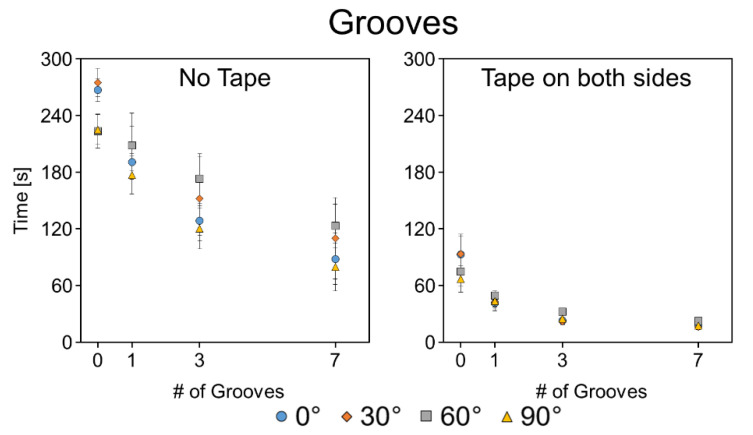
Angled wicking data for untaped and taped channels with lengthwise grooves. Data presented as mean ± SD. *n* = 5.

In addition to the uniformly etched channels, channels containing lengthwise grooves were also tested. Figure 9 shows the time it took liquid to wick the full 40 mm length of the channels at the various angles for channels containing 0, 1, 3, or 7 grooves. Unlike the uniformly etched channels, these channels show a clear trend of faster wicking with more grooves, both with and without the tape sealing. A complete set of time vs. distance plots for the grooved channels are depicted in Figure A7 and Figure A8, and *p*-values of the *t*-tests are listed in Table A3. Time-lapse GIFs of the taped and untaped grooved channels at 0° are available in the Supplementary Materials.

The single groove channel wicked very similarly to the double-sided 75% channels, while the seven-groove channel was 1.9× faster. However, once sealed in tape, the difference between the different conditions became smaller as the channels became more heavily modified; the single-sided 100%, the double-sided 95%, and the seven-groove channel all wicked at approximately the same speeds, over 13× faster than unmodified channels.

**Figure A9:**
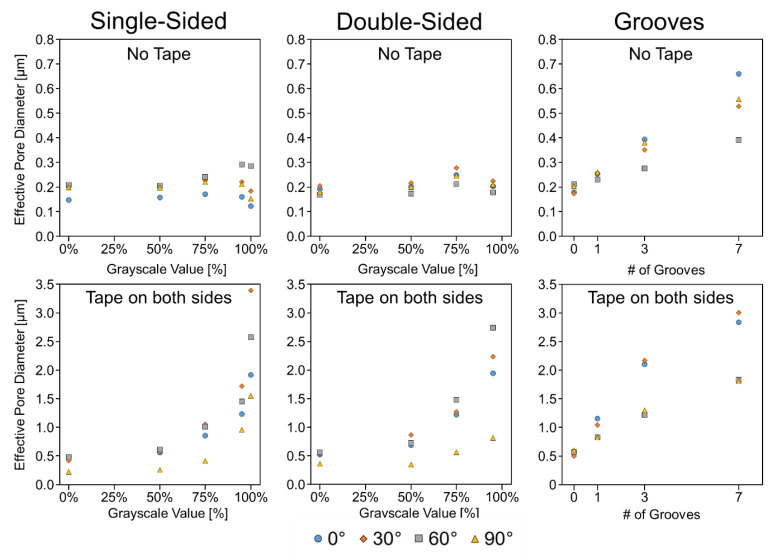
Effective pore diameters for the angled wicking channels. Averaged time vs. distance data from *n* = 5 (Figure A7 and Figure A8) were fit to the modified Lucas–Washburn model (below) described by Camplisson et al. [26] using $q_{0}$ = 1.25 × 10^−8^ m/s, derived from using an evaporative flux, *F*, of 2.5 × 10^−5^ kg/s·m^2^ from Castro et al. [10], where $F=2\times \frac{q_{0}}{\rho }$ for the untaped channels and $q_{0}$ = 0 for the taped channels.

Data in Figure A7 and Figure A8 were fitted with the modified Lucas–Washburn equation as reported by Camplisson et al. [26] in order to extract effective pore diameters (Figure A9). The effective pore diameter, $D_{c}$, is often used to characterize liquid imbibition in porous media, because, under the assumptions of the Lucas–Washburn model, permeability, k, is proportional to $D_{c}^{2}$ ($k=\phi D_{c}^{2}/32$, where ϕ is the porosity).

### 3.5. Protein Transport

**Figure 10:**
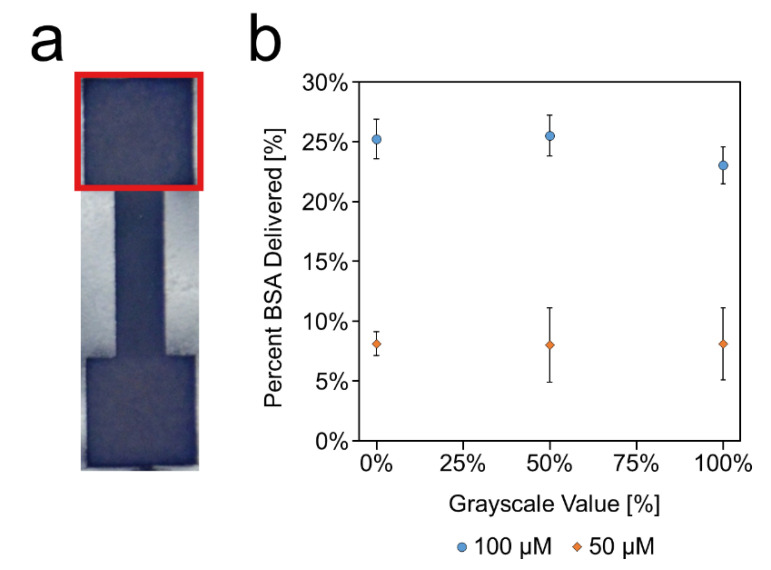
Protein transport. (**a**) Intensity of the red channel in the outlet (red square) was measured before deposition and 30 min after deposition to determine the red color shift. This value was then used to calculate the quantity of BSA-TR that made it to the outlet. (**b**) Percentage of deposited BSA-TR that wicked to the outlet of a 10 mm long, 3 mm wide channel etched at different grayscale values. Data presented as mean ± SD. *n* = 3.

To determine if the increased surface area of the more porous, etched paper would increase nonspecific binding of proteins or other analytes of interest, BSA-TR was deposited and wicked through channels etched at various levels. At the 50% etching level, the amount of BSA-TR delivered to the detection region was unchanged from that of the unetched channels, regardless of quantity of deposited BSA-TR (Figure 10). However, in the 100% channel, there was a small reduction in delivered BSA-TR in the 100 μM test, although none of the three conditions (0%, 50%, and 100%) were found to be significantly different (*p* = 0.21) according to single-factor ANOVA, α = 0.05. At lower concentrations (50 μM), there was no change in amount of BSA-TR delivered between the differently etched channels. The excess reduction in delivered BSA between 100 μM and 50 μM is due to nonspecific binding of the BSA to the cellulose [34]. However, the use of blocking agents, such as polyvinyl alcohol, are likely to be able to prevent such minor nonspecific binding [34]. Overall, these results suggest that the etched surface is unlikely to inhibit analyte transport and detection.

## 4. Conclusions

The above work details the use of laser etching as a means to control the wicking speed of liquid in paper-based microfluidic devices, where it can produce wicking speeds up to 1.1× faster and 0.9× slower than unetched paper. Wicking speeds can be further enhanced by etching both sides of the paper, with the fastest double-sided etched channels up to 1.3× faster than similarly etched single-sided equivalents. Etched channels were compared to grooved channels, with the most heavily grooved channels wicking faster than the fastest double-sided etched channel. However, upon sealing the channels in packing tape, the difference between etched and grooved channels largely disappeared, as the most heavily etched and grooved channels wicked over 13× faster than unmodified channels. The exact mechanisms behind the changes in wicking speeds as a function of etching levels are not yet well understood and require further investigation. These insights would also likely enable the engineering of custom paper with the desired wicking characteristics [35].

The etching process can be performed in conjunction with the patterning of device channels themselves, resulting in a simplified fabrication process. As a proof of concept, we demonstrated the technique’s utility in enabling sequential fluid delivery in a simple, three-legged device. In more complex devices, in addition to different etching levels in each channel, portions of individual channels can be etched to different degrees, resulting in highly tuned fluid delivery for specific applications, all without changing the overall device footprint or channel geometry. Additionally, it is likely that by combining etching with geometric patterning—such as cutting out holes in the middle of channels—a much greater range of wicking control will be available through a single fabrication step.

## Supplementary Materials

The following animation files are available online at https://www.mdpi.com/2072-666X/11/8/773/s1, Single-Sided-No-Tape-0deg.gif (single-side etched channels without tape, wicked vertically), Single-Sided-Tape-Both-0deg.gif (single-side etched channels with tape on both sides, wicked vertically), Double-Sided-No-Tape-0deg.gif (double-side etched channels without tape, wicked vertically), Double-Sided-Tape-Both-0deg.gif (double-side etched channels with tape on both sides, wicked vertically), Grooves-No-Tape-0deg.gif (grooved channels without tape, wicked vertically), Grooves-Tape-Both-0deg.gif (grooved channels with tape on both sides, wicked vertically), Demo-0-0-0.gif (demonstration device, unetched, wicked horizontally), and Demo-75-0-75.gif (demonstration device, outer channels etched, wicked horizontally).

## Appendix A

(A1) $$l\left(t\right)=\sqrt{\frac{\gamma r\phi h\cos\theta }{4\mu q_{0}}\left(1-e^{\frac{2q_{0}}{\phi h}t}\right)}$$

where the surface tension, γ, is 7.28 × 10^−2^ N/m, the porosity, ϕ, is 0.706, the thickness of the paper, *h*, is 180 µm, the contact angle, *θ*, is taken as 0°, as the cellulose paper is a fully wetting material, and the viscosity of water, *µ*, is 1 × 10^−3^ N·s/m^2^. The evaporation rate, $q_{0}$, is the volumetric evaporation rate per unit area. *r* is effective pore radius (*r* = *D_c_*/2).

**Table A1:** *p*-values for the data presented in Figure 7 (single- and double-sided etched channels without tape), generated using a two-tailed, unequal variance t-test comparing the 30°, 60°, and 90° conditions to the 0° (vertical wicking). Values below 0.05 are in bold text. Nearly all of the values in the no tape, single-sided condition have a *p*-value below 0.05 due to that condition’s vertical wicking results having considerable variability.

	**Single-Sided**					**Double-Sided**			
	0%	50%	75%	95%	100%	0%	50%	75%	95%
30°	**0.027**	**0.048**	**0.036**	**0.020**	**0.031**	0.267	0.300	0.083	0.284
60°	**0.019**	**0.036**	**0.019**	**0.004**	**0.006**	0.275	0.126	**0.045**	0.371
90°	**0.026**	0.054	**0.033**	**0.020**	0.082	0.936	0.645	0.957	0.205

**Table A2:** *p*-values for the data presented in Figure 8 (single- and double-sided etched channels with tape on both sides), generated using a two-tailed, unequal variance t-test comparing the 30°, 60°, and 90° conditions to the 0° (vertical wicking). Values below 0.05 are in bold text.

	**Single-Sided**					**Double-Sided**			
	0%	50%	75%	95%	100%	0%	50%	75%	95%
30°	0.726	0.128	**0.014**	**0.000**	**0.001**	0.748	0.427	0.682	0.252
60°	0.706	0.094	**0.049**	**0.000**	**0.003**	0.193	0.056	**0.008**	**0.008**
90°	**0.001**	**0.021**	**0.011**	0.664	0.879	**0.036**	**0.011**	0.100	**0.048**

**Table A3:** *p*-values for the data presented in Figure 9 (untaped and taped channels with lengthwise grooves), generated using a two-tailed, unequal variance *t*-test comparing the 30°, 60°, and 90° conditions to the 0° (vertical wicking). Values below 0.05 are in bold text.

	**No Tape**				**Tape Both**			
	0	1	3	7	0	1	3	7
30°	0.392	0.349	0.321	0.371	0.975	0.803	0.512	0.418
60°	**0.003**	0.122	**0.015**	0.060	0.132	**0.035**	**0.000**	**0.004**
90°	**0.002**	0.217	0.513	0.616	**0.039**	0.757	0.541	0.931
