# Supplementary figures and images for: Modifying Wicking Speeds in Paper-Based Microfluidic Devices by Laser-Etching
Source: Micromachines (Basel). 2020 Aug 14;11(8):773. doi: 10.3390/mi11080773 (PMC7463483; doi:10.3390/mi11080773)

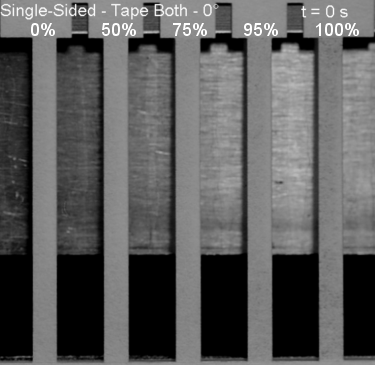

Supplement: Supplementary file 1 [file micromachines-11-00773-s001.zip › Single-Sided-Tape-Both-0deg.gif]

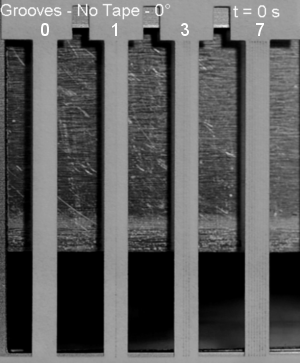

Supplement: Supplementary file 1 [file micromachines-11-00773-s001.zip › Grooves-No-Tape-0deg.gif]

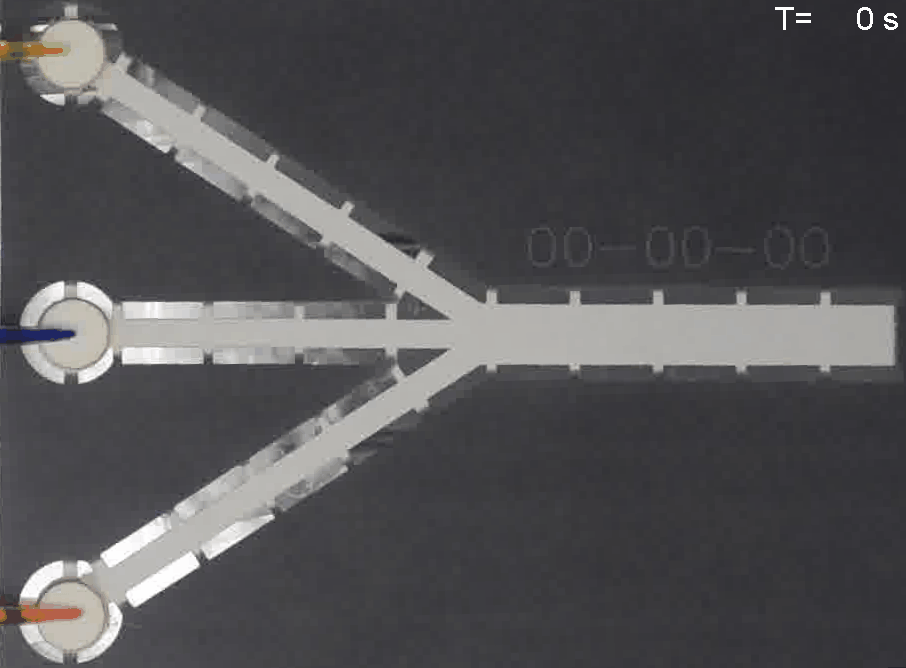

Supplement: Supplementary file 1 [file micromachines-11-00773-s001.zip › Demo-0-0-0.gif]

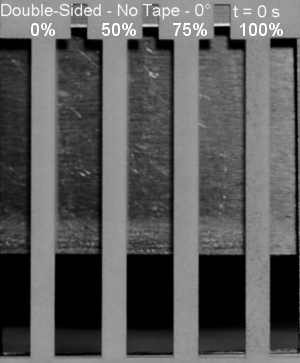

Supplement: Supplementary file 1 [file micromachines-11-00773-s001.zip › Double-Sided-No-Tape-0deg.gif]

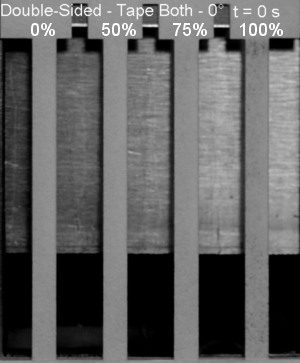

Supplement: Supplementary file 1 [file micromachines-11-00773-s001.zip › Double-Sided-Tape-Both-0deg.gif]

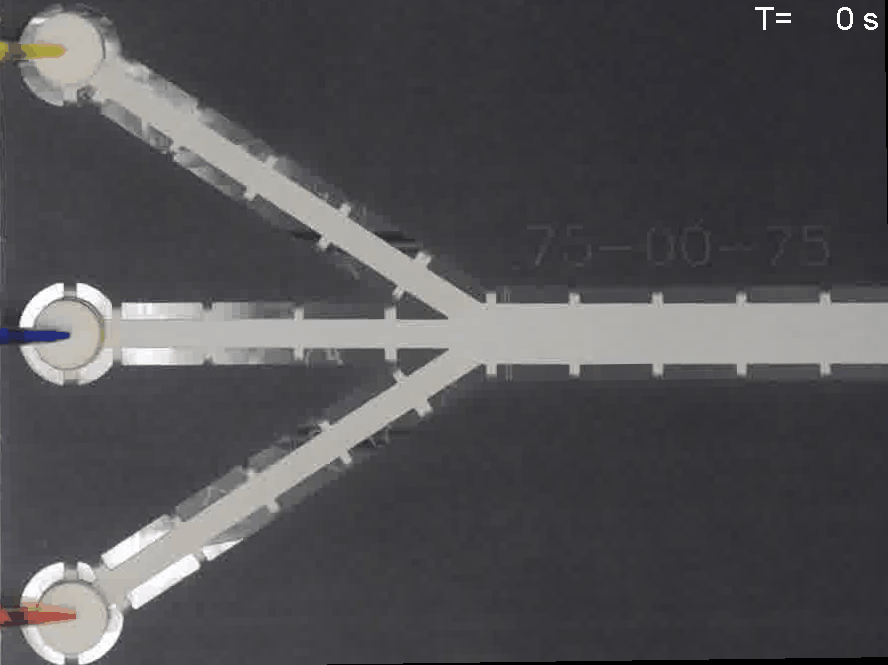

Supplement: Supplementary file 1 [file micromachines-11-00773-s001.zip › Demo-75-0-75.gif]

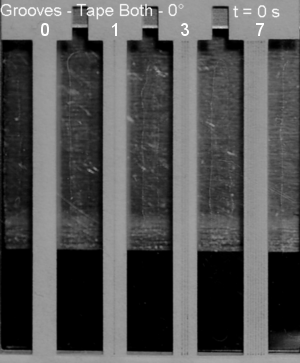

Supplement: Supplementary file 1 [file micromachines-11-00773-s001.zip › Grooves-Tape-Both-0deg.gif]

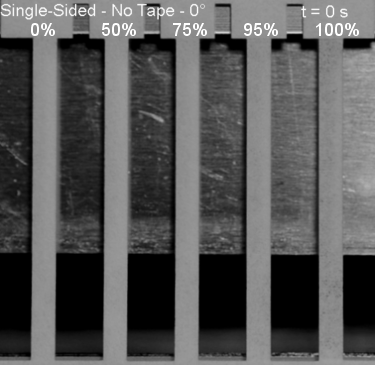

Supplement: Supplementary file 1 [file micromachines-11-00773-s001.zip › Single-Sided-No-Tape-0deg.gif]
